# Prediction of Risk Factors for Coronary Heart Disease Using Framingham Risk Score in Korean Men

**DOI:** 10.1371/journal.pone.0045030

**Published:** 2012-09-19

**Authors:** Jae-Hong Ryoo, Soo Hyun Cho, Sang-Wook Kim

**Affiliations:** 1 Department of Occupational Medicine, Kangbuk Samsung Hospital, Sungkyunkwan University, College of Medicine, Seoul, Korea; 2 Department of Preventive Medicine, College of Medicine, Kyung Hee University, Seoul, Korea; 3 Department of Family Medicine, College of Medicine, Chung-Ang University Hospital, Seoul, Korea; 4 Heart Research Institute, Chung-Ang University Hospital, Seoul, Korea; Ochsner Health System, United States of America

## Abstract

**Background:**

Currently, there is sparse data available on the relationship between coronary heart disease (CHD) and its risk factors estimated by the Framingham Risk Score (FRS) in Korea. This is particularly true when looking at risk factors of CHD associated with the FRS after adjustment for other covariates especially in healthy subjects.

**Methodology/Principal Findings:**

We conducted a prospective cohort study to examine the association between the risk factors of CHD and the risk for CHD estimated by FRS in 15,239 men in 2005 and 2010. The FRS is based on six coronary risk factors: gender, age, total cholesterol, high-density lipoprotein (HDL)-cholesterol, systolic blood pressure (BP), and smoking habit. Multiple linear regression analysis was used to analyze the relationships between the FRS and risk factors for CHD. This study reported that apolipoproetein B (apoB), apoA-I, apoB/apoA-I, alcohol intake, log-transformed TG, log-transformed hsCRP, LDL-cholesterol, hypertension, diabetes, regular exercise, and BMI were significantly associated with the FRS. Above all, the partial *R*-square of apoB was 14.77%, which was overwhelmingly bigger than that of other variables in model V. This indicated that apoB accounted for 14.77% of the variance in FRS.

**Conclusion/Significance:**

In this study, apoB was found to be the most important determinant for the future development of CHD during a 5-year follow-up in healthy Korean men.

## Introduction

Diabetes mellitus, hypertension, and dyslipidemia have been regarded as established predictors of cardiovascular disease. Life style risk factors including dietary habits, physical inactivity, smoking, alcohol intake, stress, and obesity are strongly associated with established cardiovascular risk factors and cardiovascular disease [1]–[3].

Apolipoproteins are important structural and functional proteins in lipoprotein particles, which transport lipids. Apolipoproteins also regulate the synthesis and metabolism of lipoprotein particles and stabilize their structure [4]. Measurement and calculation of the apolipoprotein B (apoB) and apoB/apoA-I ratio may improve the prediction of risk for cardiovascular disease, as it represents the balance between proatherogenic and antiatherogenic lipoproteins [5].

The reduction of risk factors for cardiovascular disease can prevent the incidence of the cardiovascular diseases [6]. There are many studies and programs on the prevention of cardiovascular risk. Estimates of cardiovascular risk in healthy population are usually calculated from risk prediction models derived from prospective and observational studies [7]–[9]. The Framingham Risk Score (FRS) is a traditionally used algorithm in primary prevention strategies for the assessment of 10-year risk for coronary heart disease (CHD) events in middle-aged, asymptomatic individuals [7], [10]. Recently, several studies have reported that other risk factors for CHD were associated with the FRS [11], [12].

However, as far as we know, there is sparse data available on the relationships between CHD and its risk factors estimated by the FRS in Korea and especially how the risk factors for CHD is associated with the FRS after adjustment for other covariates in particularly healthy subjects. Therefore, the present study examines the risk factors related with the FRS in healthy Korean men. We also intend to identify which is the most important contribution to the risk of CHD estimated by FRS.

## Materials and Methods

### Study design and subjects

**Figure 1:**
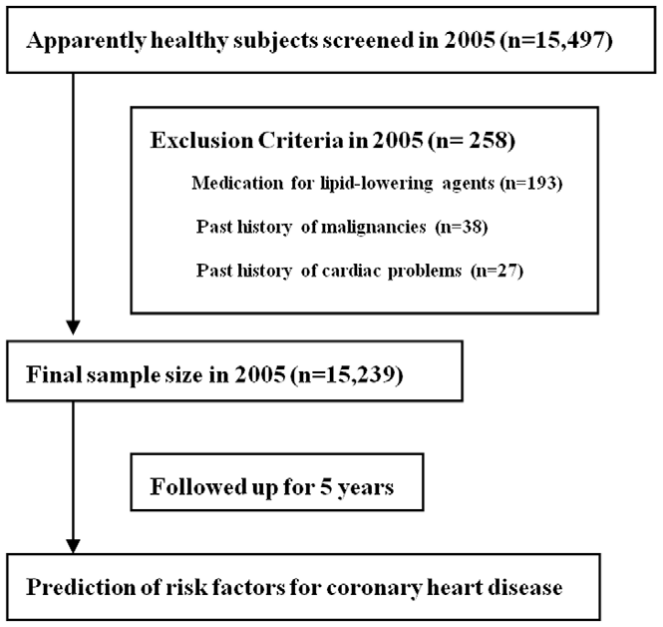
Selection of study participants.

A prospective cohort study was conducted to examine the risk factors for CHD in healthy Korean men who were employed at various companies in Korea. All employees participated in an annual health examination, as is required by Korea's Industrial Safety and Health law. The study population consisted of individuals who had comprehensive health examinations at baseline (2005) and were reexamined 5 year later (2010) at Kangbuk Samsung Hospital. Initially 15,497 individuals were identified. Among the initial 15,497 individuals, 258 were excluded for various reasons: 193 were taking lipid-lowering medication at their initial examinations; 38 had past histories of malignancies; 27 had past histories of cardiac problems (angina and myocardial infarction). After these exclusions, 15,239 men were enrolled in this analysis and were observed for relationships between risk factors for CHD and FRS (Figure 1). Each participant provided written informed consent when checking the questionnaire and ethics approvals for the study protocol. Analysis of the data was obtained from the institutional review board of College of Medicine, Chung-Ang University Hospital (Ethics Committee reference number C2011125(575)) in accordance with the Declaration of Helsinki principles.

### Clinical and laboratory measurements

Initial health examinations that were performed in 2005 included medical histories, physical examinations, questionnaires about health-related behavior, anthropometric measurements, and biochemical measurements. Medical history and history of drug prescription was assessed by the examining physicians. All the participants were asked to respond to a questionnaire on health-related behavior. Questions about alcohol intake included the frequency of alcohol consumption on a weekly basis and the usual amount that was consumed on a daily basis (≥20 g/day). We considered persons reporting that they smoked at that time to be current smokers. In addition, the participants were asked about their weekly frequency of physical activity, such as jogging, bicycling, and swimming that lasted long enough to produce perspiration (≥1 time/week). Hypertension was defined as either taking antihypertensive agents or having blood pressure of ≥140/90 mmHg. Diabetes was defined by current use of blood glucose–lowering agents or fasting blood glucose level of ≥126 mg/dL.

Blood samples were collected after more than 12 hours of fasting and were drawn from an antecubital vein. The serum levels of fasting glucose, total cholesterol, triglyceride (TG), low-density lipoprotein (LDL) cholesterol, and high-density lipoprotein (HDL) cholesterol were measured using the Bayer Reagent Packs on an automated chemistry analyzer (ADVIA 1650™ Autoanalyzer, Bayer HealthCare Ltd., Tarrytown, NY, USA). High-sensitivity C-reactive protein (hsCRP) was measured by immunonephelometry (Dade Behring Co., Marburg, Germany), and apoA-I and apoB was measured by rate nephelometry (Beckman Instruments, Fullerton, CA, USA). Distribution of values was assessed by the Kolmogorov-Smirnov test for normality of distribution. Because the values of hsCRP and TG were skewed to the right, its log-transformed data were used in multiple linear regression analysis.

Trained nurses obtained sitting blood pressure (BP) levels with a standard mercury sphygmomanometer. The first and fifth Korotkoff sounds were used to estimate the systolic BP and the diastolic BP. Height and weight were measured after an overnight fast with the subjects wearing lightweight hospital gowns and no shoes. Body mass index (BMI) was calculated as weight (kg) divided by the height (m) squared.

### Framingham risk score assessment

The FRS was calculated from the National Cholesterol Education Program (NCEP) Adult Treatment Panel(ATP) III algorithm, based on six coronary risk factors: gender, age, total cholesterol, HDL-cholesterol, systolic BP and smoking habit [10]. Among these factors, age, BP, and cholesterol levels were categorized according to their values and smoking status was classified as either “current smoker” or “non-smoker”. Finally, the corresponding point was assigned to each individual and the total score was used as the individual's CHD risk level.

### Statistical analysis

The distributions of continuous variables were evaluated, and transformations were used in the analysis as required. The Pearson correlation analysis was used to show the correlation coefficient (*β*) between the FRS and clinical variables. Multiple linear regression analysis was used to analyze the relationships between the FRS with apoB, apoA-I, apoB/apoA-I, alcohol intake, log-transformed TG, log-transformed hsCRP, LDL-cholesterol, hypertension, diabetes, regular exercise, and BMI, which were included in the regression model for adjustment from model I to model V. The statistical analysis for the data was performed with SPSS version 17.0 (SPSS Inc.). All the reported *P* values were two-tailed, and those <0.05 were considered to be statistically significant.

## Results

The relevant clinical characteristics of the 15,239 male subjects are shown in Table 1. Overall, the mean age and BMI were 44.0±5.7 years and 24.4±2.7 kg/m^2^, respectively (mean ± S.D.). The mean apoB and apoA-I were 100.2±22.4 mg/dL and 138.8±21.8 mg/dL, respectively (mean ± S.D.). Table 2 shows the correlation coefficient (*β*) between the FRS and the clinical variables. The correlation coefficient (*β*) of apoB is 0.38, which is relatively bigger than that of apoA-I (β = −0.08). All the clinical variables except for apoA-I, HDL-cholesterol, and regular exercise showed a positively significant correlation with the FRS. ApoA-I (*r* = −0.08, *P*<0.001), HDL-cholesterol (*r* = −0.14, *P*<0.001) and regular exercise (*r* = −0.03, *P* = 0.001) were negatively correlated with the FRSs respectively. Table 3 and 4 show multiple linear regression analysis of the FRS as for independent variables in model I, II, III, IV and V. Through the correlation analysis, apoB, apoA-I, apoB/apoA-I, alcohol intake, log-transformed TG, log-transformed hsCRP, LDL-cholesterol, hypertension, diabetes, regular exercise, and BMI were selected. Model I consisted of important covariates: alcohol intake, log-transformed TG, log-transformed hsCRP, LDL-cholesterol, hypertension, diabetes, regular exercise, and BMI which accounted for 16.28% of the variance in FRS. Log-transformed TG was the main determinant of FRS (partial *R* ^2^ = 8.89%). Model II consisted of model I plus apoA-I. The partial *R* ^2^ of apoA-I was 0.47%. When entering apoB/apoA-I into the regression analysis of model I (model III), the main determinant was changed into apoB/apoA-I. The partial *R* ^2^ of apoB/apoA-I and log-transformed TG were 12.67% and 2.59%, respectively. When entering apoB into the regression analysis of model I (model IV), apoB was the main determinant, instead of log-transformed TG. The partial *R* ^2^ of apoB and log-transformed TG were 14.77% and 1.77%, respectively. Finally, all variables including apoA-I, apoB/apoA-I, and apoB were entered as explanatory (model V). This showed that apoB survived and proved to be the main determinant of FRS (partial *R* ^2^ = 14.77%). The final model was more informative (adjusted *R* ^2^ = 18.90%).

**Table 1:** Baseline Characteristics of the study subjects (n = 15,239).

Age (years)	44.0±5.7
≤39	3,377 (22.1)
40–49	9,824 (64.5)
≥50	2,038 (13.4)
BMI (kg/m^2^)	24.4±2.7
Systolic BP (mmHg)	114.7±14.3
Diastolic BP (mmHg)	77.5±9.6
Total cholesterol (mg/dL)	195.6±31.9
Triglyceride (mg/dL)	149.2 (93.0–181.0)
HDL-cholesterol (mg/dL)	49.7±10.1
LDL-cholesterol (mg/dL)	115.0±26.8
Fasting serum glucose (mg/dL)	98.0±15.6
ApoA-I (mg/dL)	138.8±21.8
ApoB (mg/dL)	100.2±22.4
ApoB/ApoA-I	0.7±0.2
hsCRP (mg/L)	0.1 (0.0–0.1)
Current smoker	6,120 (40.9)
Alcohol intake	1,783 (11.9)
Regular exercise	2,371 (15.8)
Diabetes	606 (4.0)
Hypertension	2,790 (18.3)

All values are the mean ± SD, median (interquartile range) or the number of subjects (percent of the total).

**Table 2:** Correlation between the FRS and the clinical variables.

Variable	Correlation coefficient(*β*)	*P*-value*
Age	0.49	<0.001
BMI	0.15	<0.001
Systolic BP	0.09	<0.001
Diastolic BP	0.12	<0.001
Total cholesterol	0.29	<0.001
Triglyceride	0.25	<0.001
HDL-cholesterol	−0.14	<0.001
LDL-cholesterol	0.26	<0.001
Fasting serum glucose	0.10	<0.001
ApoA-I	−0.08	<0.001
ApoB	0.38	<0.001
ApoB/ApoA-I	0.35	<0.001
hsCRP	0.03	0.001
Current smoking (No = 0, Yes = 1)	0.38	<0.001
Alcohol intake (No = 0, Yes = 1)	0.09	<0.001
Regular exercise (No = 0, Yes = 1)	−0.03	0.001
Hypertension (No = 0, Yes = 1)	0.13	<0.001
Diabetes (No = 0, Yes = 1)	0.06	<0.001

* *P*-value by Pearson correlation analysis.

**Table 3:** Multiple linear regression analysis of the FRS as for important covariates (model I to model III).

	MODEL I				MODEL II				MODEL III			
Variable	Parameter estimate	Standard error	Partial *R* ^2^ (%)	*P*-value*	Parameter estimate	Standard error	Partial *R* ^2^ (%)	*P*-value*	Parameter estimate	Standard error	Partial *R* ^2^ (%)	*P*-value*
Alcohol intake (No = 0, Yes = 1)	0.776	0.097	0.47	<0.001	0.921	0.099	0.47	<0.001	0.950	0.097	0.74	<0.001
Log(TG)	1.852	0.067	8.89	<0.001	1.818	0.067	8.89	<0.001	1.351	0.074	2.59	<0.001
LDL-cholesterol	0.031	0.001	5.32	<0.001	0.031	0.001	5.32	<0.001	0.014	0.001	0.45	<0.001
Hypertension (No = 0, Yes = 1)	0.789	0.083	0.89	<0.001	0.841	0.083	0.89	<0.001	0.839	0.082	0.98	<0.001
Log(hsCRP)	0.216	0.032	0.30	<0.001	0.191	0.032	0.22	<0.001	0.156	0.032	0.14	<0.001
Regular exercise (No = 0, Yes = 1)	−0.540	0.088	0.28	<0.001	−0.584	0.088	0.32	<0.001	−0.564	0.088	0.29	<0.001
Diabetes (No = 0, Yes = 1)	0.577	0.162	0.09	<0.001	0.600	0.162	0.09	<0.001	0.531	0.161	0.08	0.001
BMI	−0.030	0.013	0.04	0.020	−0.040	0.013	0.07	0.002	−0.046	0.013	0.09	0.001
ApoA-I					−0.011	0.001	0.47	<0.001				
ApoB/ApoA-I									3.684	0.237	12.67	<0.001
Model *R*-square (%)			16.28				16.74				18.02	

* *P*-value by multiple linear regression analysis.

MODEL II = MODEL I plus apoA-I. MODEL III = MODEL I plus apoB/apoA-I.

**Table 4:** Multiple linear regression analysis of the FRS as for important covariates (model IV and model V).

	MODEL IV				MODEL V			
Variable	Parameter estimate	Standard error	Partial *R* ^2^ (%)	*P*-value*	Parameter estimate	Standard error	Partial *R* ^2^ (%)	*P*-value*
Alcohol intake (No = 0, Yes = 1)	0.653	0.096	0.38	<0.001	0.800	0.097	0.54	<0.001
Log(TG)	0.789	0.092	1.77	<0.001	0.741	0.092	1.77	<0.001
LDL-cholesterol	−0.012	0.003	0.16	<0.001	−0.012	0.003	0.17	<0.001
Hypertension (No = 0, Yes = 1)	0.695	0.082	0.70	<0.001	0.752	0.082	0.70	<0.001
Log(hsCRP)	0.180	0.032	0.20	<0.001	0.150	0.032	0.12	<0.001
Regular exercise (No = 0, Yes = 1)	−0.442	0.087	0.21	<0.001	−0.484	0.087	0.24	<0.001
Diabetes (No = 0, Yes = 1)	0.382	0.161	0.04	0.012	0.404	0.160	0.05	0.016
BMI	−0.033	0.013	0.05	0.017	−0.043	0.013	0.08	0.001
ApoA-I					0.003	0.003	0.00	0.343
ApoB/ApoA-I					2.430	0.262	0.46	<0.001
ApoB	0.064	0.004	14.77	<0.001	0.047	0.004	14.77	<0.001
Model *R*-square (%)			18.29				18.90	

* *P*-value by multiple linear regression analysis.

MODEL IV = MODEL I plus apoB. MODEL V = MODEL I plus apoA-I, apoB/apoA-I and apoB.

## Discussion

In this present study, a significant positive correlation between the serum levels of apoB and the FRS was observed in healthy Korean men. Through the multiple linear regression model I∼V, this study also showed that apoB was the most important determinant amongst the risk of CHD estimated by FRS. In model I, TG and LDL-cholesterol which had been known important risk factor of CHD was the main determinant of FRS (partial *R*-square = 8.89 and 5.32%). Model IV and V showed that apoB proved to be the main determinant, instead of TG, LDL-cholesterol, and apoB/apoA-I. There was multicollinearity between apoB and LDL-cholesterol because apoB was highly correlated with LDL-cholesterol (*r* = 0.85, *P*<0.001). Irrespective of this weakness, we planned to examine the risk factors for CHD and were able to identify the fact that the partial *R* ^2^ for apoB was overwhelmingly bigger than that of other risk factors. We also made this analysis without LDL-cholesterol variables and were able to reach similar results (data not shown).

Almost all of the protein component of LDL-cholesterol is made up of ApoB, and is also a component of chylomicrons, very low-density lipoprotein cholesterol and its metabolic remnants, and lipoprotein (a) [13]. Conversely, apoA-I is important in removing excess cholesterol from tissues and incorporating it into HDL-cholesterol for reverse transport to the liver, thus manifesting antiatherogenic effects [13].

In the western countries, high apoB and low apoA-I have been shown to be an independent predictor of CHD [14]–[22]. However, there is scarce longitudinal evidence to date showing that apolipoproteins have a predictive value for CHD in Korea or in other Asian countries. In this regard, the present findings suggest the possibility that apoB rather than apoA-I and apo B/apoA-I may be associated with the prediction of the prospective development of CHD in a healthy Korean population.

The reasons and mechanisms for why apoB is highly associated with CHD are not fully understood. Barter and his colleagues emphasized that apoB is a better indicator of the total number of atherogenic particles because each very-low-density lipoprotein, intermediate-density lipoprotein, LDL-cholesterol, and lipoprotein (a) particles contain only one molecule of apoB-100 [23].

ApoB also assembles the precursors of LDL in the liver including the primordial particle, VLDL1, and VLDL2. ApoB is also responsible for delivering lipids from the liver and intestine to peripheral tissue [24], [25]. Total apoB levels reflect the entire spectrum of pro-atherogenic particles, including intermediate-density lipoprotein, very-low-density lipoprotein, and LDL-cholesterol whereas LDL-cholesterol levels does not [26].

Previous studies have reported conflicting results about which lipid measure is the best predictor of future incidence of CHD. Several recent reports have raised the possibility that the apolipoproteins have been proposed as a better index for predicting the risk of cardiovascular disease than the traditional lipid parameters for CHD, based on the premise that apoB levels better reflect the number of atherogenic lipoprotein particles in a given volume of plasma [14], [15], [20], [21], [27], whereas other studies do not support this notion [16]–[18], [22].

Our study, however, has some limitations. First off, our study is confined to a relatively homogeneous group of Korean male of individuals who were recruited at a single urban hospital, and therefore, we cannot confidently apply our results to other racial groups. Another factor to consider is the fact our sample consisted of westernized Koreans living in urban environments so the results from this study may not be an accurate reflection on the whole Korean population. Therefore, further research on the compatibility of the FRS scale for Korean people may be necessary. Additionally, the participants were self-selected, so this study may show participant selection bias. Regardless of these limitations, strengths of the present study include the large sample size of healthy male participants with comprehensive measurements of a panel of lipids and apolipoproteins, including the direct measurement of standard lipids. Additionally, detailed information on cardiovascular risk factors was available. Although numerous studies have been published, the cause-and-effect relationships cannot be confirmed due to the cross-sectional nature of the studies. As a result, this study is considered to provide strong evidence for correlations between FRS and the other various lipid parameters, such as the apoB, apoA-I, etc.

## Conclusion

We have showed that apoB rather than apoA-I and apoB/apoA-I was the most important contribution to the risk of CHD estimated by the FRS indicating that apoB might play a crucial role in the risk of CHD. The findings of the present study have clinically important implications. ApoB measurements have been standardized and are easily accomplished with an automated assay, so it may be more convenient [28]. Furthermore, fasting samples are not required, which is a clear advantage over LDL-cholesterol measuring methods [28].

The cost for apoB measurement is equivalent to those for the total costs of cholesterol and HDL-cholesterol measurements [29]. The present diagnostic guideline of dyslipidemia did not include the apolipoprotein level in clinical settings. In the future, the proper guideline for diagnose, treatment, and medication should also be established.
